# A Live Attenuated AP2X-1-Deficient *Toxoplasma* Strain Confers Protective Immunity Against Acute and Chronic Toxoplasmosis in a Murine Model

**DOI:** 10.3390/ani16162453

**Published:** 2026-08-07

**Authors:** Wen-Bo Hao, Li-Xiu Sun, Ru-Shi Tu, Zhi Zheng, Chen-Ran Tian, Xing-Quan Zhu, Xiao-Nan Zheng

**Affiliations:** 1 Shanxi Key Laboratory of Animal Disease Research, Prevention and Control, College of Veterinary Medicine, Shanxi Agricultural University, Jinzhong 030801, China; 16696739873@163.com (W.-B.H.); tuss44@163.com (R.-S.T.); 15863201790@163.com (Z.Z.); chenrantian2019@163.com (C.-R.T.); 2 Department of Microbiology and Immunology, School of Basic Medical Sciences, Dali University, Dali 671000, China; sunlixiu1026@163.com

**Keywords:** *Toxoplasma gondii*, PruΔ*ap2X-1*, live-attenuated vaccine, Th1 immune response, acute and chronic infection

## Abstract

*Toxoplasma gondii* is an obligate intracellular apicomplexan parasite that poses serious risks to immunocompromised individuals and the global livestock industry. Our previous study showed that AP2X-1 regulates stage conversion and virulence of *T. gondii*; however, whether AP2X-1-deficient strains can serve as live-attenuated vaccine candidates is unknown. Here, we evaluated the in vivo virulence, protective efficacy, and induced immunity of the *ap2X-1* knockout strain PruΔ*ap2X-1*. In Kunming mice, infection with even the highest tested dose (5 × 10^6^ tachyzoites) of PruΔ*ap2X-1* resulted in 100% survival without detectable brain cysts. Vaccination with 10^6^ PruΔ*ap2X-1* tachyzoites conferred complete protection (100% survival) against acute challenge with homologous type II Pru or heterologous ToxoDB#9 PYS strains, whereas control mice exhibited only 0% to 16.7% survival. Moreover, immunized mice orally challenged with Pru cysts showed 100% survival, and no brain cysts were detected under the conditions tested, compared with only 30% survival in control mice challenged with 10 cysts and no survival with 40 cysts. The vaccination induced a Th1-biased response as evidenced by significantly elevated *T. gondii*-specific total IgG, IgG2a, and the Th1 cytokines IFN-γ and IL-2, along with a significantly reduced IgG1/IgG2a ratio. Collectively, the attenuated PruΔ*ap2X-1* strain generates strong protective immunity against acute and chronic infection with low-virulence strains (the type II Pru and ToxoDB#9 PYS), as well as partial protection against the hypervirulent type I RH strain, positioning it as a potential candidate for a live-attenuated vaccine, with a favorable safety profile observed under the experimental conditions.

## Simple Summary

Toxoplasmosis, caused by the zoonotic parasite *Toxoplasma gondii*, is a major concern for immunocompromised individuals and the livestock industry. Research has shown that AP2X-1 is important for growth, conversion between tachyzoites and bradyzoites, and sexual stage development of *T. gondii*. In the present study, we found that all mice infected with the PruΔ*ap2X-1* knockout strain survived even at the highest dose (5 × 10^6^ tachyzoites), and no brain cysts were detected. Vaccinated mice were fully protected against both acute disease caused by the type II Pru and ToxoDB#9 PYS strains, and chronic infection induced by tissue cysts, whereas vaccinated mice had modestly extended survival time without conferring complete protection against challenge with the highly virulent RH strain. Moreover, vaccination induced a strong Th1-biased cellular and humoral immune response. Thus, PruΔ*ap2X-1* represents a potential live-attenuated vaccine candidate against toxoplasmosis, with a favorable safety profile observed under the experimental conditions.

## 1. Introduction

*Toxoplasma gondii* is a globally distributed obligate intracellular protozoan capable of infecting almost all warm-blooded animals, including humans, making toxoplasmosis an important zoonotic parasitic disease [1]. Although most immunocompetent individuals remain asymptomatic, infection poses serious risks to immunocompromised populations and pregnant women, and constitutes a major constraint on the global livestock industry [2,3,4]. Although the species comprises only a single described taxon, its genetic architecture is highly complex, with approximately 300 distinct genotypes identified to date; among these, types I, II, and III constitute the three major clonal lineages, which exhibit markedly divergent virulence profiles in mice [2,5,6]. ToxoDB#9 is the predominant *T. gondii* genotype prevailing in animals in China [7].

The complex multistage life cycle of *T. gondii*, its broad range of intermediate hosts, and its capacity to effectively evade the host immune system collectively pose substantial challenges to the control of this zoonotic parasite [8,9]. Although the combination of pyrimethamine and sulfadiazine represents the most effective current treatment for acute toxoplasmosis, pharmacotherapy is frequently associated with adverse effects and fails to eliminate tissue-sequestered bradyzoites, rendering it incapable of eradicating chronic infection. In addition, concerns over drug residues further limit its clinical application [10]. The development of safe and effective toxoplasmosis vaccines has become a research priority. Recent efforts have tested multiple platforms, including DNA/RNA vaccines, subunit vaccines, nanoparticle delivery systems, and live recombinant or genetically attenuated strains [11,12].

Among these, live-attenuated *T. gondii* strains have shown protective efficacy in animal models. For instance, TS-4 immunization fully protects mice against lethal type I challenge [13]. The oocyst-deficient T-263 mutant reduces fecal oocyst shedding in cats after challenge [14]. In livestock, the S48 strain lowers parasite loads in mutton and decreases abortion rates in pregnant ewes [15]. These observations highlight live-attenuated vaccines as a promising avenue for toxoplasmosis control, as they closely emulate natural infection and efficiently prime adaptive immunity [16]. Even so, important drawbacks remain. The S48 strain carries a potential risk of mutation and reversion to virulence, and the commercial vaccine derived from it, Toxovax^®^, is licensed in only a few countries [15]. Despite these advances, existing live vaccines remain incapable of completely eliminating tissue cysts, underscoring a persistent gap in the effective control of toxoplasmosis [17]. Addressing this gap remains a critical scientific priority.

In recent years, the rapid advancement and application of genetic engineering technologies have increasingly enabled the construction of live attenuated vaccine strains through targeted disruption of key virulence or metabolism-related genes in *T. gondii*. The immunoprotective efficacy of such mutants has been validated in various mouse models. For example, immunization with the PruΔ*gra72* strain elicits immune protection against acute and chronic toxoplasmosis in a murine model [18]. Similarly, vaccination of mice with the WH3Δ*rop18* and PruΔ*pp2a-c* strains induced strong protection against acute and chronic toxoplasmosis of mice [19,20].

Among the key regulators of stage conversion and virulence are the AP2 transcription factors, a family of proteins characterized by plant-like AP2 DNA-binding domains that orchestrate stage-specific gene expression [21]. Several members of this family have been functionally characterized in regulating stage conversion, cyst formation, or virulence [22,23,24,25,26]. Among these, AP2X-1 has emerged as a particularly critical regulator. Genetic ablation of *ap2X-1* impairs tachyzoite invasion and intracellular replication, induces upregulation of bradyzoite and sexual stage-specific genes, and markedly suppresses in vivo virulence and brain cyst formation. Mechanistically, AP2X-1 recruits the HDAC3/MORC complex to silence transcriptional programs required for bradyzoite differentiation and sexual development [27]. Owing to these essential functions, the AP2X-1 deletion mutant displays substantially reduced virulence at 5 × 10^3^ and 5 × 10^5^ tachyzoites, and shows an impaired capacity to establish chronic infection, positioning it as a promising live-attenuated candidate [27]. Nevertheless, studies evaluating the immunoprotective potential of the AP2X-1-deficient strain against toxoplasmosis remain limited.

In the present study, we systematically assessed the vaccine efficacy of the PruΔ*ap2X-1* strain, characterized the immune responses it elicited, and determined its protective effects against acute and chronic *T. gondii* infection, thus offering experimental evidence that supports the further development of AP2X-1-based live attenuated vaccine against toxoplasmosis.

## 2. Materials and Methods

### 2.1. Mice and Parasites

This study utilized 6–8-week-old female Kunming mice, an outbred strain widely used in preclinical vaccine evaluations. The genetic diversity of outbred populations better mimics the heterogeneous responses of diverse hosts, offering a potentially more rigorous assessment of vaccine efficacy [28]. All Kunming mice were acquired from Beijing Sibefu Biotechnology Co., Ltd. (Beijing, China) and kept in a specific-pathogen-free environment (25 °C, 50–60% humidity) with ad libitum access to food and water. Following a one-week acclimatization to the facility, experimental procedures were initiated. Every effort was made to comply with animal welfare regulations and to minimize any potential distress to the animals. All animal procedures were performed in accordance with the Institutional Animal Care and Use Committee of Shanxi Agricultural University (Approval No.: SXAU-EAW-2020ME8.OJ.005018326).

The parasite strains used in this study included the type I RH strain, the type II Pru strain, the ToxoDB#9 PYS strain, the background strain PruΔ*ku80*Δ*hxgprt*, and the previously gene-disrupted strain PruΔ*ku80*Δ*hxgprt*Δ*ap2X-1* (PruΔ*ap2X-1*) [27]. Tachyzoites were propagated in human foreskin fibroblasts (HFFs; SCRC-1041; American Type Culture Collection, Manassas, VA, USA) cultured in DMEM (Gibco, Suzhou, China) supplemented with 2% fetal bovine serum (FBS, Gibco, Melbourne, VIC, Australia) at 37 °C, as detailed elsewhere [18]. Type II Pru cysts were harvested from the brains of infected mice according to a published method [20].

### 2.2. Optimization of the Immunization Dose of PruΔap2X-1

To evaluate the in vivo virulence of the PruΔ*ap2X-1* strain, 6–8-week-old female Kunming mice (*n* = 6 per group) received an intraperitoneal injection of escalating doses of PruΔ*ap2X-1* (5 × 10^4^, 10^5^, 5 × 10^5^, 10^6^, or 5 × 10^6^ tachyzoites) or wild-type Pru (5 × 10^3^, 5 × 10^4^, 10^5^, 5 × 10^5^, 10^6^, or 5 × 10^6^ tachyzoites). The presence of clinical toxoplasmosis symptoms and mortality were recorded twice per day for 30 days post-infection (dpi) in all infected mice. Upon completion of the experiment (day 30), we counted the brain cysts in all surviving animals. Specifically, whole brains were harvested, individually homogenized in 1.5 mL PBS, and 10 µL aliquots of the homogenate were examined microscopically. Cysts were counted in 10 replicate aliquots per sample, and the average count was used to calculate the total number of cysts per brain.

### 2.3. Measurement of T. gondii-Specific Antibodies in Mouse Sera

For immunization, mice received an intraperitoneal injection of either 10^6^ PruΔ*ap2X-1* tachyzoites or an equal volume of phosphate-buffered saline (PBS) as a mock-immunized control. Humoral immune responses in PruΔ*ap2X-1*-immunized mice were assessed at 45 days post-vaccination (dpv) using serum samples from the immunized and control animals (*n* = 5 per group). Total IgG, IgG1, and IgG2a were measured by enzyme-linked immunosorbent assay (ELISA), using soluble *T. gondii* antigen (STAg) and commercial antibodies, prepared and used as previously described [18]. Briefly, 96-well plates were coated with STAg, and diluted sera were added after blocking. Detection was performed with HRP-conjugated goat anti-mouse secondary antibodies: anti-mouse IgG (1:3000; Abcam, AB97040, Cambridge, UK), anti-mouse IgG1 (1:5000; Abcam, AB98693, UK), and anti-mouse IgG2a (1:5000; Abcam, AB98698, UK), followed by 3,3′,5,5′-Tetramethylbenzidine (TMB; Beyotime Biotech, P0209-100mL, Shanghai, China) development and absorbance measurement at 450 nm.

### 2.4. Measurement of Cytokines in Splenocyte Supernatants from Immunized and Control Mice

To evaluate cellular immune responses, splenocytes were aseptically collected from immunized and control mice (*n* = 5 per group), and cytokine levels in culture supernatants were measured as described previously [18]. Following washing and gentle disruption of splenic tissue on a 200-mesh nylon sieve, the homogenate was cleared by centrifugation (1500× *g*, 10 min). Red blood cells were removed through 3 min exposure to lysis buffer, leaving a pure splenocyte fraction that was resuspended in RPMI 1640 (Solarbio, Beijing, China) supplemented with 10% FBS. Trypan blue staining confirmed that >95% of cells were viable. The suspension was normalized to 3 × 10^6^ cells/mL and stimulated with STAg at 10 μg/mL. Supernatants were harvested at predetermined intervals for cytokine measurement: 24 h (IL-2, IL-4), 72 h (IL-10), and 96 h (IL-12, IFN-γ). Levels of cytokines were assayed using ELISA kits according to the suppliers’ instructions: IL-2 (BioLegend, 431007, San Diego, CA, USA), IL-4 (BioLegend, 431107, USA), IL-10 (BioLegend, 431417, USA), IL-12 (BioLegend, 433607, USA), and IFN-γ (BioLegend, 430807, USA).

### 2.5. Protection Against Acute and Chronic Infection

At 45 dpv, the protective efficacy against acute toxoplasmosis was evaluated by intraperitoneally challenging immunized and control mice (*n* = 6 per group) with 10^2^ or 10^3^ tachyzoites of the RH or PYS strain, or with 10^6^ tachyzoites of the Pru strain. Next, we assessed protection against chronic infection by challenging separate groups of immunized and control mice (*n* = 10 per group) orally with 10 or 40 Pru cysts at 45 dpv. At 30 dpi, brain cysts were enumerated microscopically in mice that survived chronic infection. Brains without visible cysts were further examined by semi-nested PCR targeting the *B1* gene as previously described [29]. Each PCR reaction was performed in duplicate, with positive controls (Pru strain tachyzoite genomic DNA) and negative controls (no-template control) included in each run.

### 2.6. Statistical Analysis

All experiments were performed once with the indicated group sizes (*n* = 6 per group for virulence and acute protection experiments, *n* = 10 per group for chronic protection experiments, and *n* = 5 per group for antibody and cytokine measurements). Statistical analyses were performed using GraphPad Prism 10.1.2 (GraphPad Software, Inc., La Jolla, CA, USA). Prior to analysis, data normality was assessed using the Shapiro–Wilk test, and homogeneity of variance was assessed using the F-test as appropriate. Antibody data between immunized and control mice were analyzed using Welch‘s *t*-test due to unequal variances, whereas IgG1 and IgG2a levels within the immunized group were compared using a paired *t*-test. The IgG1/IgG2a ratio between groups was assessed using an unpaired Student’s *t*-test. For cytokine data, which were not consistently normally distributed across analytes, group comparisons were performed using the Mann–Whitney U test. For multiple-groups comparisons involving brain cyst data, the Kruskal–Wallis test was used, followed by Dunn’s multiple comparison test with *p*-value adjustment. Survival curves were estimated using the Kaplan–Meier method and compared using the Log-rank (Mantel–Cox) test, including the calculation of hazard ratios (HRs) and 95% confidence intervals (CIs). Exact *p*-values are reported where possible. All statistical tests were two-tailed, and differences with *p* < 0.05 were considered statistically significant.

## 3. Results

### 3.1. Assessment of Attenuated Virulence and Optimization of Vaccination Dose in Mice

AP2 transcription factors exhibit diverse functions, acting as molecular switches that either activate or repress gene cohorts involved in cell cycle progression, stage conversion between tachyzoites and bradyzoites, and parasite virulence [24,30,31]. Deletion of certain AP2 genes in type II strains attenuates parasite virulence [24,26,27]. The previously generated knockout strain PruΔ*ap2X-1* was used in this study [27].

**Figure 1:**
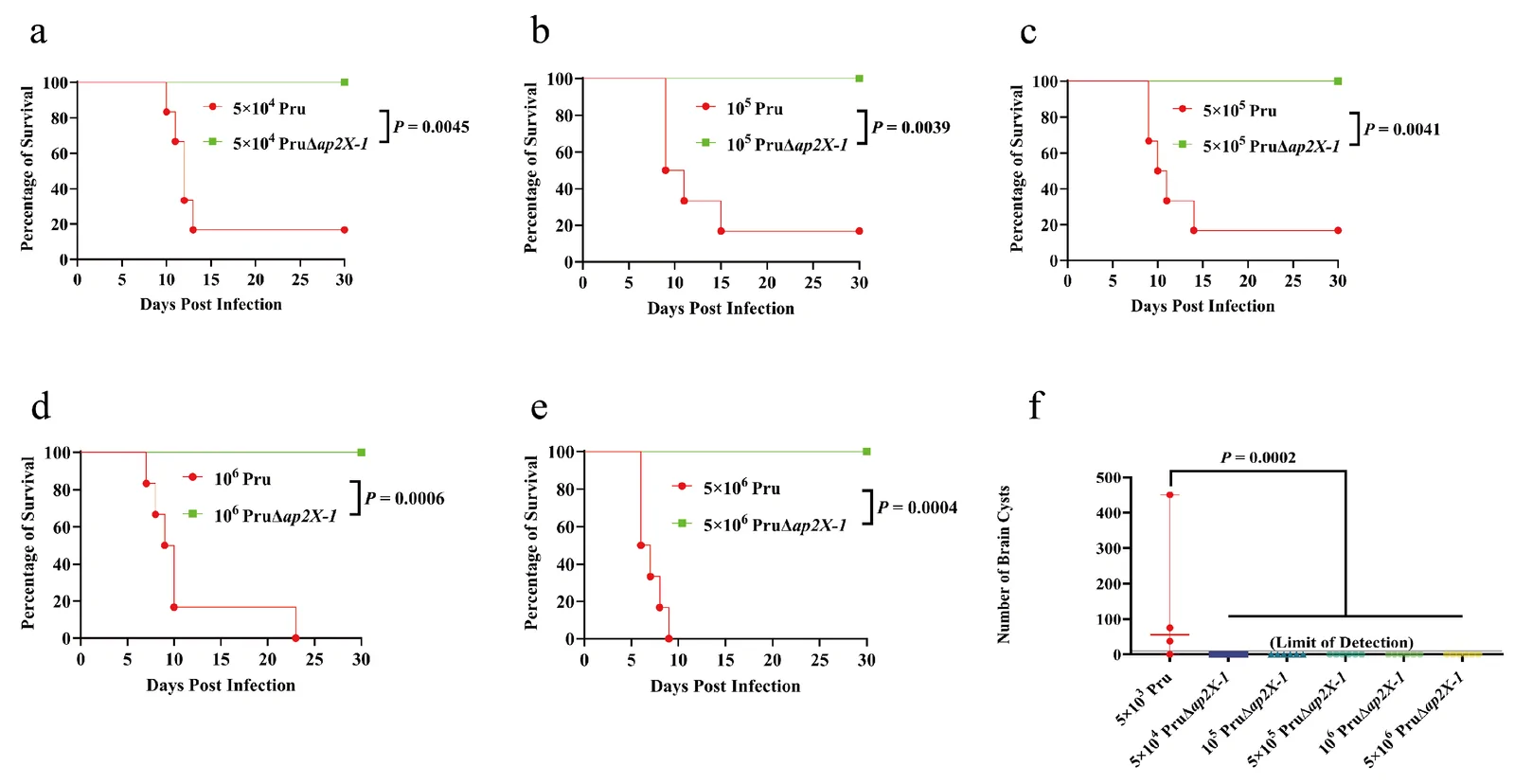
Assessment of PruΔ*ap2X-1* virulence in mice. (**a**–**e**) Survival curves of Kunming mice following intraperitoneal inoculation with escalating doses (5 × 10^4^, 10^5^, 5 × 10^5^, 10^6^, or 5 × 10^6^) of either PruΔ*ap2X-1* or wild-type (Pru) tachyzoites (*n* = 6 per group). Survival curves were compared using the Log-rank (Mantel–Cox) test. PruΔ*ap2X-1* significantly reduced the mortality risk compared with the wild-type Pru strain at each dose tested, with hazard ratios (HR) for mortality risk and pairwise *p* values as follows: 5 × 10^4^, HR = 14.78 (95% CI: 2.305–94.83), *p* = 0.0045; 10^5^, HR = 17.29 (95% CI: 2.491–120.0), *p* = 0.0039; 5 × 10^5^, HR = 15.36 (95% CI: 2.379–99.19), *p* = 0.0041; 10^6^, HR = 21.55 (95% CI: 3.732–124.5), *p* = 0.0006; and 5 × 10^6^, HR = 26.94 (95% CI: 4.317–168.1), *p* = 0.0004. HR > 1 indicates an increased mortality risk in wild-type Pru-infected mice relative to PruΔ*ap2X-1*-infected mice. (**f**) Brain cyst burden in surviving mice at 30 days post-infection, including data from mice infected with 5 × 10^3^ wild-type tachyzoites (initial *n* = 6 per group). Each symbol represents an individual mouse, and cyst counts were compared using the Kruskal–Wallis test (overall *p* = 0.0002), followed by Dunn‘s post hoc test with *p*-value adjustment. All mice infected with PruΔ*ap2X-1* showed significantly lower cyst counts than the control mice infected with the wild-type Pru strain (adjusted *p* = 0.0003 for each comparison).

**Figure 2:**
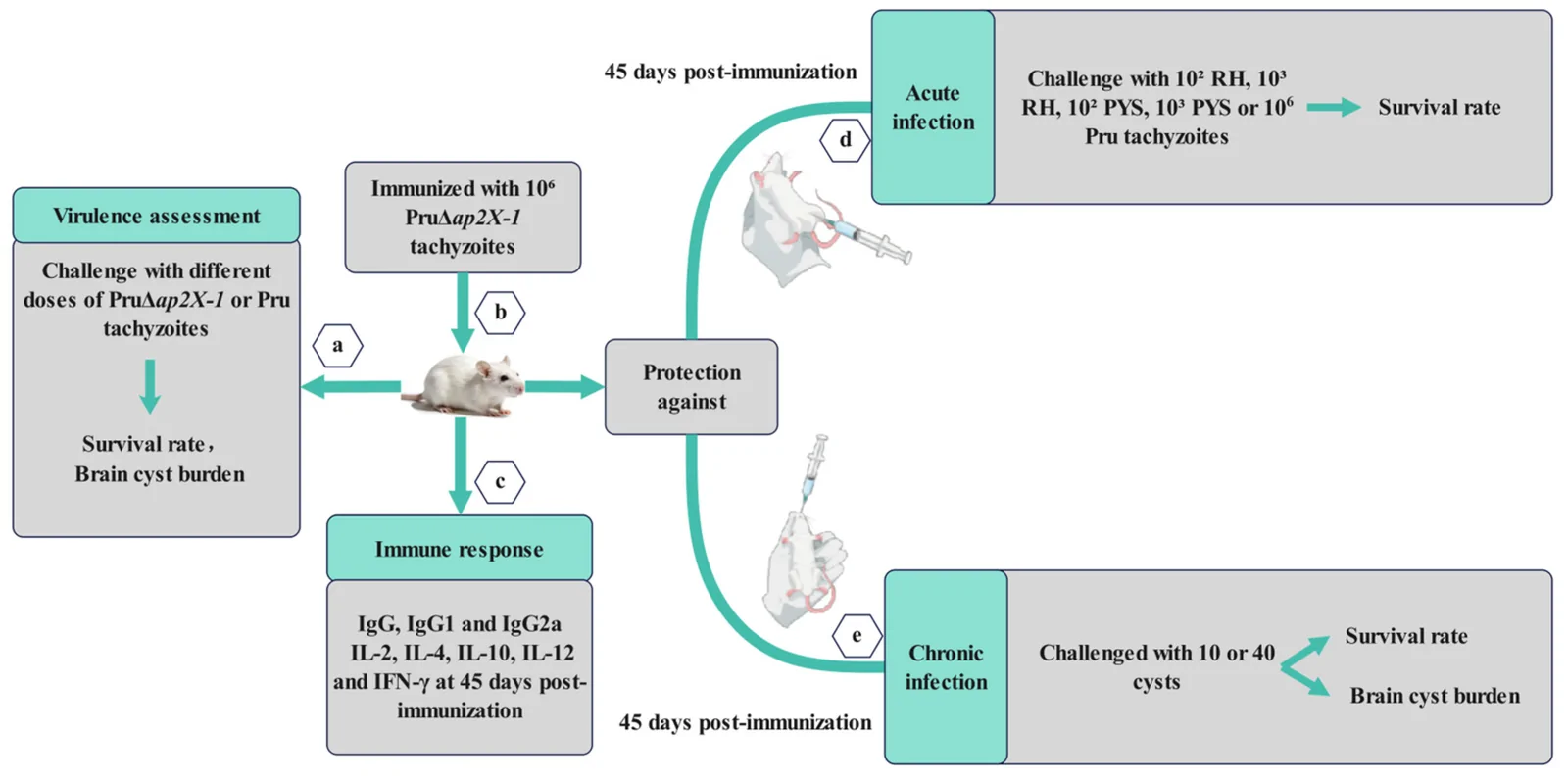
Schematic overview of the experimental design to evaluate PruΔ*ap2X-1* as a live-attenuated vaccine candidate. (**a**) Virulence assessment of PruΔ*ap2X-1*. (**b**) Immunization with PruΔ*ap2X-1* tachyzoites. (**c**) Detection of immune responses. (**d**) Protection assessment against acute infection with *T. gondii* tachyzoites. (**e**) Protection assessment against chronic infection with Pru cysts.

The virulence of PruΔ*ap2X-1* was evaluated by intraperitoneal inoculation of Kunming mice with escalating doses of either PruΔ*ap2X-1* or wild-type Pru tachyzoites. All mice receiving the highest dose of the knockout strain (5 × 10^6^ tachyzoites) survived to the end of the observation period. By contrast, infection with 10^6^ or 5 × 10^6^ wild-type Pru tachyzoites was uniformly lethal. At intermediate doses (5 × 10^4^, 10^5^, and 5 × 10^5^ Pru tachyzoites), only 1 of 6 mice per group (16.7%) survived (Figure 1a–e). Survival curve comparisons revealed that PruΔ*ap2X-1* significantly reduced the mortality risk compared with wild-type Pru at each dose tested, with hazard ratios ranging from 14.78 to 26.94. Aside from mild ruffled fur observed in mice receiving the highest dose (5 × 10^6^) of the knockout strain, PruΔ*ap2X-1*-inoculated mice exhibited no obvious clinical signs. Among mice that survived a challenge with 5 × 10^3^ wild-type tachyzoites, the median number of brain cysts was 56.25; by contrast, no cysts were detected microscopically in the brains of any mouse that received PruΔ*ap2X-1*, regardless of the inoculated dose (Figure 1f). Collectively, these results indicate that PruΔ*ap2X-1* is markedly attenuated in mice. Based on these dose–response data, a dose of 10^6^ tachyzoites was selected for subsequent immunization experiments. This dose was well tolerated in the dose-escalation study and was chosen with reference to immunization doses used in previous live-attenuated *T. gondii* vaccine studies, including PruΔ*pp2a-c* and RHΔ*ompdc*Δ*uprt* [20,32]. A schematic overview of the experimental design is presented in Figure 2.

### 3.2. Immunization Elicits a Strong and Specific Immune Response

**Figure 3:**
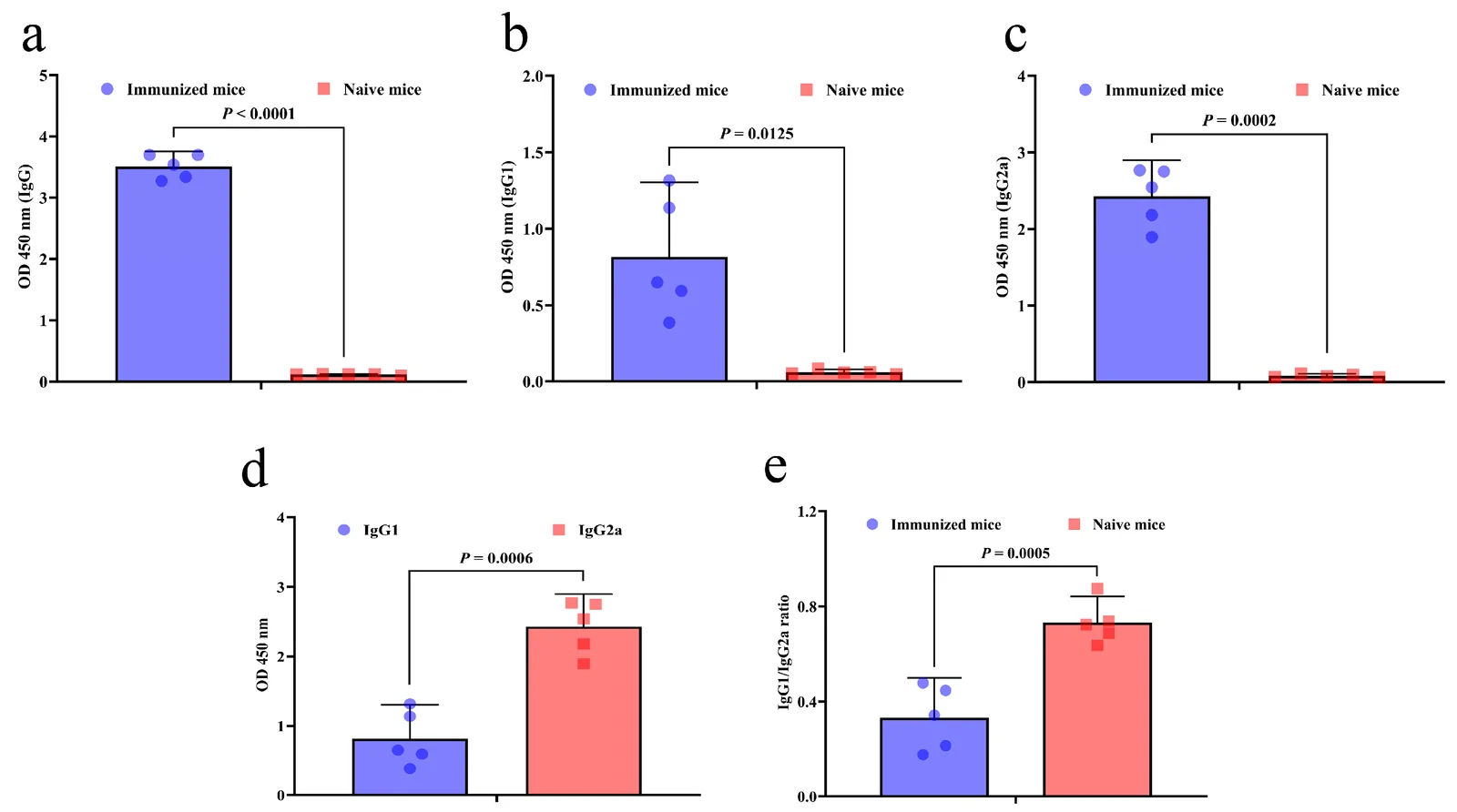
PruΔ*ap2X-1* immunization elicits humoral immune responses in mice. (**a**–**c**) Serum levels of *T. gondii*-specific total IgG, IgG1, and IgG2a levels in immunized versus control mice (*n* = 5 per group). (**d**) IgG1 versus IgG2a within the immunized group. (**e**) IgG1/IgG2a ratio in immunized versus control mice. Data are shown as individual values with mean ± 95% CI. *p* values were calculated using Welch’s *t*-test (**a**–**c**), paired *t*-test (**d**), or unpaired Student’s *t*-test (**e**). IgG, *p* < 0.0001; IgG1, *p* = 0.0125; IgG2a, *p* = 0.0002; IgG1 versus IgG2a, *p* = 0.0006; ratio, *p* = 0.0005.

At 45 dpv, splenocytes and serum samples were collected from immunized and control mice to evaluate humoral and cellular immune responses (Figure 2c). Compared with controls, PruΔ*ap2X-1*-immunized mice exhibited significantly higher levels of *Toxoplasma*-specific total IgG, as well as IgG1 and IgG2a (Figure 3a–c). Specifically, the mean total IgG level in immunized mice was 3.507, representing an approximately 30-fold increase compared with controls (0.115; *p* < 0.0001). IgG2a levels showed a similar 29-fold elevation (2.427 vs. 0.083; *p* = 0.0002), while IgG1 levels increased by approximately 14-fold (*p* = 0.0125). These results demonstrate that PruΔ*ap2X-1* immunization elicits a robust humoral response.

In the immunized mice, IgG2a levels were significantly and consistently greater than those of IgG1 (*p* = 0.0006, Figure 3d), yielding an IgG1/IgG2a ratio of 0.34 ± 0.13, The ratio was significantly lower than that of the control mice (0.73 ± 0.09, *p* = 0.0005, Figure 3e), representing a 54% reduction. Notably, the ratio values of the two groups were completely non-overlapping, with all immunized mice exhibiting ratios below 0.5 and all control mice above 0.6, confirming a consistent and robust Th1-biased humoral response induced by PruΔ*ap2X-1* immunization.

**Figure 4:**
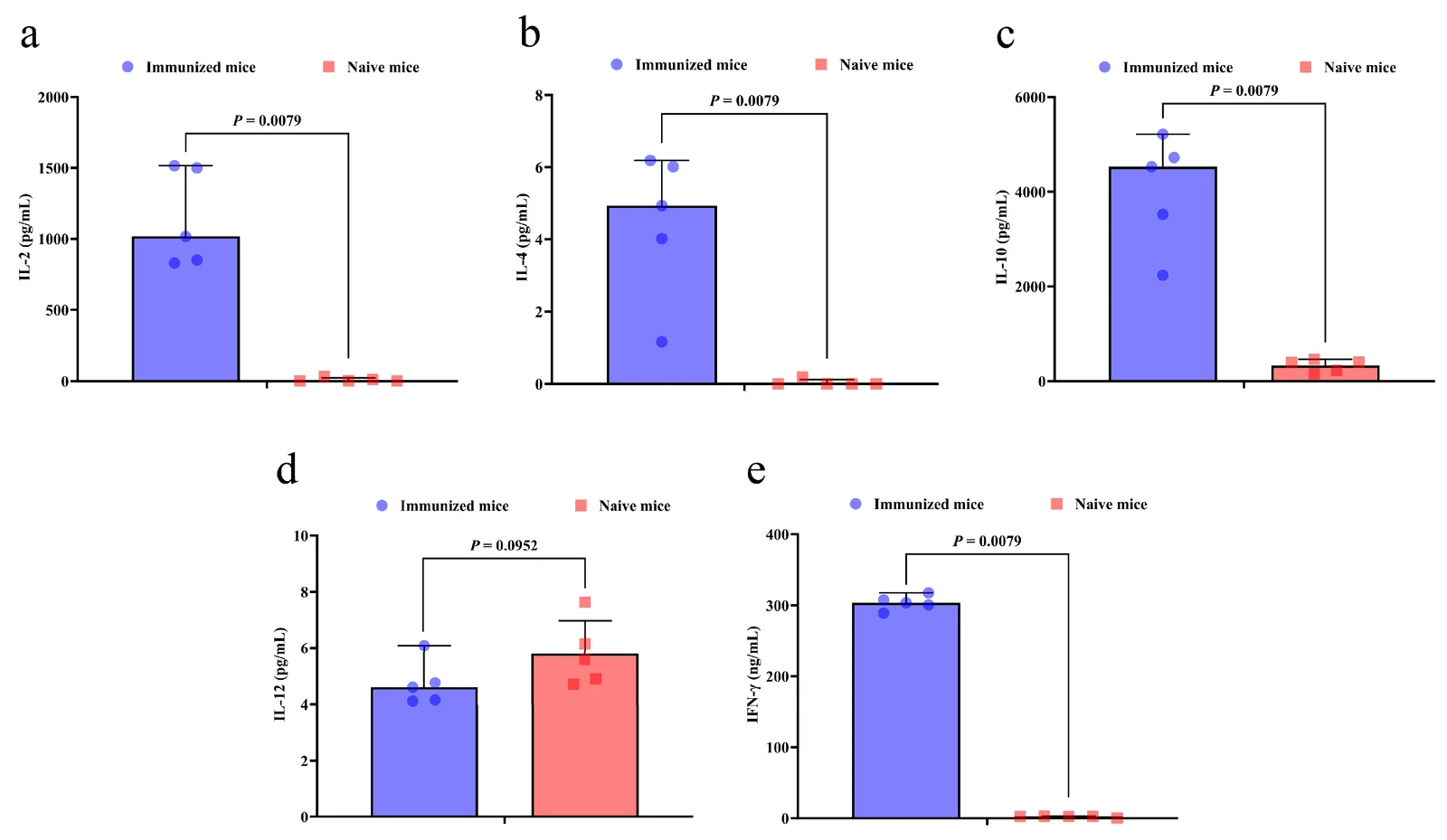
Cytokine responses in immunized versus control mice at 45 days post-vaccination (dpv). (**a**–**e**) Concentrations of IL-2, IL-4, IL-10, IL-12, and IFN-γ in culture supernatants of STAg-stimulated splenocytes from immunized and control mice (*n* = 5 per group). Data are shown as individual values with median ± 95% CI. *p* values were calculated using the Mann–Whitney U test. IL-2, *p* = 0.0079; IL-4, *p* = 0.0079; IL-10, *p* = 0.0079; IL-12, *p* = 0.0952; IFN-γ, *p* = 0.0079.

To further explore vaccination-elicited cellular immunity, splenocytes were isolated from immunized and control mice at 45 dpv. After in vitro culture and STAg stimulation, cytokine levels in the supernatants were determined by ELISA (Figure 2c). Compared with control mice, PruΔ*ap2X-1*-immunized mice exhibited significantly elevated production of IL-2 (116-fold), IFN-γ (136-fold), IL-4 (117-fold), and IL-10 (12-fold) (all *p* < 0.01); whereas IL-12 levels remained unaltered (Figure 4). Among all cytokines examined, IFN-γ was the most prominently induced Th1-associated cytokine, with a mean concentration of 303.54 ng/mL. Notably, IFN-γ levels significantly exceeded those of IL-2, IL-4, IL-10, and IL-12 (Figure S1a,d), and the markedly high IFN-γ/IL-4 ratio (approximately 67,990) supports the polarization toward a Th1-biased response. This cytokine profile is consistent with the Th1-biased humoral immune response observed in the antibody analysis. We next evaluated its protective efficacy against acute and chronic challenge infections.

### 3.3. Protection of PruΔap2X-1 Vaccination Against Acute Infection with Tachyzoites of Different T. gondii Genotypes in Mice

At 45 dpv, we assessed the protective efficacy of PruΔ*ap2X-1* vaccination against acute *T. gondii* infection by challenging both vaccinated and control Kunming mice with either 10^2^ or 10^3^ tachyzoites of the ToxoDB#9 PYS strain, 10^2^ or 10^3^ tachyzoites of the virulent type I RH strain, or 10^6^ tachyzoites of the type II Pru strain (Figure 2d).

**Figure 5:**
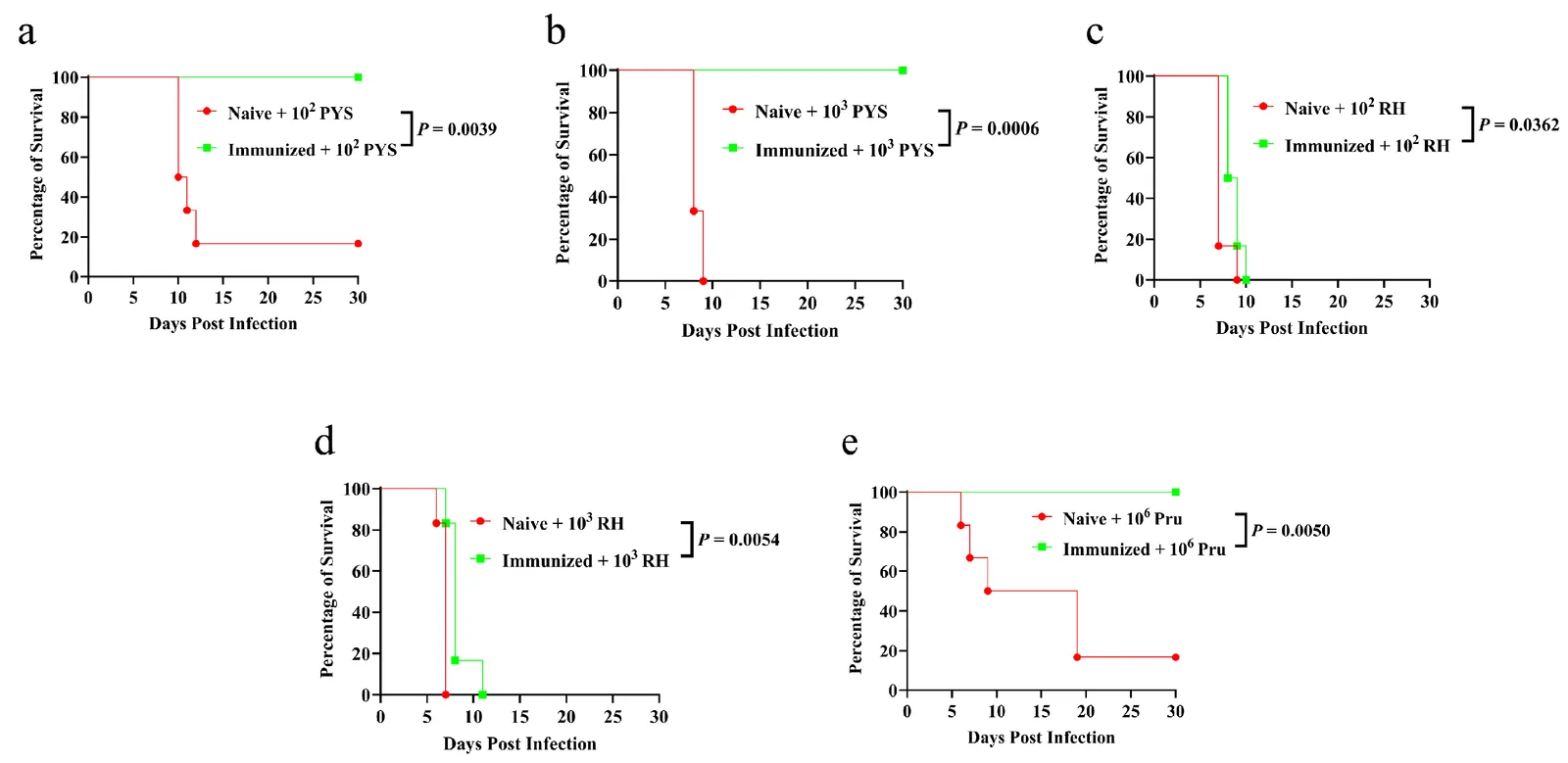
Protection conferred by PruΔ*ap2X-1* immunization against acute *T. gondii* infection at 45 days post-vaccination (dpv). Survival curves of immunized and control mice (initial *n* = 6 per group) challenged with the indicated doses of tachyzoites: 10^2^ PYS (**a**), 10^3^ PYS (**b**), 10^2^ RH (**c**), 10^3^ RH (**d**), or 10^6^ Pru (**e**). Survival was monitored for 30 days. Survival curves were compared using the Log-rank (Mantel–Cox) test. Hazard ratios (HR) for mortality risk in control mice relative to immunized mice, with 95% confidence intervals and *p* values, are as follows: 10^2^ PYS, HR = 17.29 (95% CI: 2.491–120.0), *p* = 0.0039; 10^3^ PYS, HR = 28.15 (95% CI: 4.152–190.8), *p* = 0.0006; 10^2^ RH, HR = 6.624 (95% CI: 1.130–38.84), *p* = 0.0362; 10^3^ RH, HR = 16.28 (95% CI: 2.279–116.3), *p* = 0.0054; 10^6^ Pru, HR = 14.06 (95% CI: 2.219–89.12), *p* = 0.0050. HR > 1 indicates an increased mortality risk in control mice relative to immunized mice.

After challenge with 10^2^ or 10^3^ PYS tachyzoites, all immunized mice survived. However, only 16.7% of controls survived when challenged with 10^2^ PYS tachyzoites, and no control mice survived after challenge with 10^3^ PYS tachyzoites (Figure 5a,b). Survival curve comparisons revealed that immunization with PruΔ*ap2X-1* significantly reduced the mortality risk compared with control mice challenged at both PYS doses. Following challenge with the virulent RH strain, although immunization with PruΔ*ap2X-1* resulted in a modest extension of median survival time compared with control mice, all mice in both groups ultimately died within 12 days post-challenge during the 30-day observation period (Figure 5c,d). In contrast, immunization provided complete protection against 10^6^ Pru tachyzoites, with all immunized mice surviving compared with only 16.7% survival in the control group (Figure 5e).

Collectively, these results demonstrate that immunization with PruΔ*ap2X-1* confers robust protection against acute toxoplasmosis caused by low-virulence strains (PYS and Pru) and delayed mortality against the highly virulent RH strain.

### 3.4. PruΔap2X-1 Vaccination Protects Mice Against Chronic Infection Induced by Cyst Challenge

**Figure 6:**
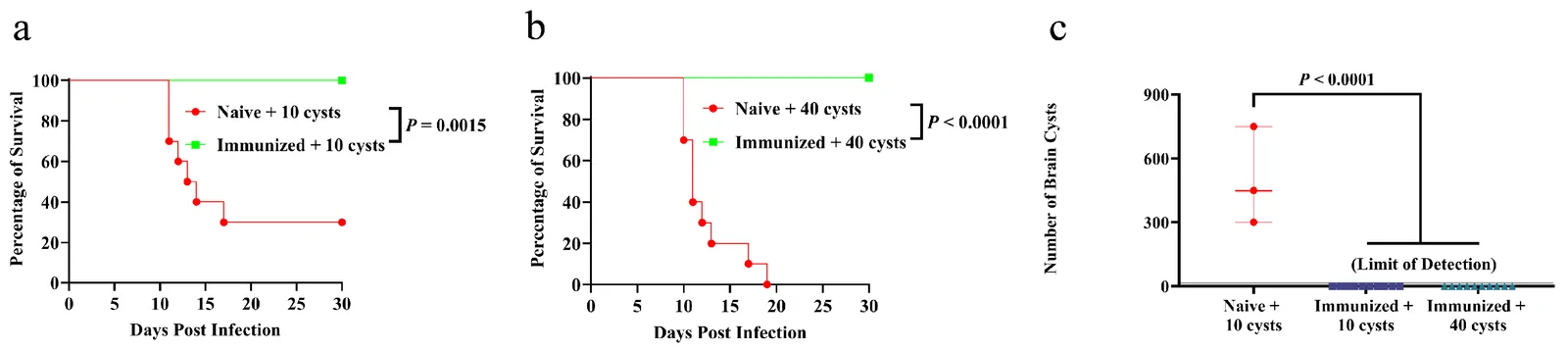
Protection conferred by PruΔ*ap2X-1* immunization against chronic *T. gondii* infection at 45 dpv. (**a**,**b**) Mice received an oral challenge with 10 (**a**) or 40 (**b**) Pru cysts (initial *n* = 10 per group). Survival was monitored for 30 days. Survival curves were compared using the Log-rank (Mantel–Cox) test. Hazard ratios (HR) for mortality risk in control mice relative to immunized mice, with 95% confidence intervals and *p* values, are as follows: 10 cysts, HR = 13.48 (95% CI: 2.86–64.30), *p* = 0.0015; 40 cysts, HR = 29.91 (95% CI: 7.23–123.5), *p* < 0.0001. HR > 1 indicates an increased mortality risk in control mice relative to immunized mice. (**c**) Brain cyst burden in surviving mice at 30 days post-challenge. Each symbol represents an individual mouse. Cyst counts were compared using Kruskal–Wallis test followed by Dunn‘s post hoc test with *p*-value adjustment (adjusted *p* < 0.0001 for each comparison).

To expand our assessment beyond acute infection, we next evaluated the protective efficacy of PruΔ*ap2X-1* vaccination against chronic infection. Immunized and control mice received an oral challenge with either 10 or 40 brain cysts of the Pru strain at 45 dpv (Figure 2e). Results showed that all vaccinated animals survived both cyst doses, yielding 100% survival (Figure 6a,b). In contrast, control mice exhibited dose-dependent lethal outcomes. In the low-dose group, mice began to show clinical signs at 7 dpi, including ruffled fur and reduced appetite, with mortality beginning on day 9, resulting in a final survival rate of 30% (Figure 6a). In the high-dose group, clinical signs appeared earlier and were more severe, and all mice succumbed to infection (Figure 6b). Taken together, these findings demonstrate that vaccination with PruΔ*ap2X-1* confers robust protection against chronic *T. gondii* infection.

### 3.5. PruΔap2X-1 Immunization Reduces Brain Cyst Burden in Mice with Chronic Infection

Brains were collected from all mice that survived for 30 days in the cyst challenge groups, and *T. gondii* cyst loads were determined. In control mice challenged with the low cyst dose (10 cysts), three mice survived, with a median brain cyst count of 450. All control mice receiving the higher cyst dose (40 cysts) died, precluding any assessment of their brain cyst burden (Figure 6c). In contrast, no tissue cysts were detected by microscopic examination in the brains of immunized mice challenged with either the low or high dose of Pru cysts. Notably, the *B1* gene was undetectable by semi-nested PCR in brain tissues from immunized mice, indicating that parasite DNA was below the detection limit (Figure S2 and Table S1). Collectively, these data indicate that vaccination with PruΔ*ap2X-1* tachyzoites markedly blocks the development of mature cysts in mice chronically infected with *T. gondii*.

## 4. Discussion

This work investigated the vaccine potential of the PruΔ*ap2X-1* knockout strain against toxoplasmosis. The results demonstrate marked in vivo attenuation of this *T. gondii* mutant in mice. Notably, vaccination with PruΔ*ap2X-1* conferred potent protection against acute infection elicited by relatively low-virulence strains (the type II Pru strain and the ToxoDB#9 PYS strain), as well as against chronic infection resulting from oral challenge with tissue cysts. Furthermore, the vaccine elicited a robust Th1-biased adaptive immune response, comprising both humoral and cellular arms. These findings establish PruΔ*ap2X-1* as a promising candidate for a live-attenuated vaccine against toxoplasmosis, contributing to the growing portfolio of genetically engineered vaccine candidates enabled by advances in gene editing technologies.

AP2X-1 has been identified as a negative regulator of sexual development in *T. gondii*, functioning via its interaction with the HDAC3/MORC complex to silence genes specific to bradyzoites and sexual stages [27]. Through its engagement with this complex, AP2X-1 acts as a transcriptional brake that prevents bradyzoite differentiation and sexual commitment. In line with this repressive role, loss of *ap2X-1* triggers spontaneous tachyzoite-to-bradyzoite conversion under standard culture conditions [27]. In the present study, mice maintained 100% survival at the highest tested dose of 5 × 10^6^ tachyzoites of the PruΔ*ap2X-1* strain, exhibiting only mild clinical signs, and no detectable brain cysts were observed under the conditions tested. This safety profile is consistent with the attenuated phenotypes reported for other AP2-family-related knockout strains, such as PruΔ*ap2XI-2* [24], further supporting the potential of targeting stage-regulating transcription factors as a rational strategy for generating safe and immunogenic live attenuated vaccines.

Immunization with PruΔ*ap2X-1* provided complete protection against challenge with relatively low-virulence strains, including the type II Pru and ToxoDB#9 PYS strains, as evidenced by 100% survival of immunized mice following challenge. When challenged with the highly virulent type I RH strain, vaccinated mice exhibited extended survival time compared with controls; however, all animals ultimately died from the infection, indicating that the protection was partial rather than complete. This differential efficacy is likely attributable to several type I-specific virulence traits that collectively impair the protective immunity elicited by the type II-based vaccine. The highly virulent ROP18/ROP5 alleles of the RH strain efficiently inactivate IFN-γ-inducible IRG proteins to evade innate clearance, a capacity largely absent in type II strains [33], while type I ROP16 suppresses proinflammatory responses via STAT3/STAT6 activation [34]. Additionally, type I GRA15 has substantially weaker NF-κB-activating activity than its type II counterpart, which normally promotes a Th1-biased inflammatory environment through NF-κB activation [35]. Together, these effector differences are thought to compromise the Th1-mediated protection elicited by the type II-background vaccine.

The incomplete cross-protection observed against the RH strain highlights a major limitation of this vaccine candidate and underscores the challenge of achieving broad cross-protection against genetically divergent strains. This limitation is not unique to the PruΔ*ap2X-1* strain; similar incomplete heterologous protection has been reported for other live attenuated vaccine candidates, including PruΔ*gra72*, ME49Δ*cdpk3*, and PruΔ*pp2a-c* [18,20,36], suggesting that the genetic divergence between type I and type II lineages poses a common hurdle for cross-strain vaccine efficacy. The incomplete heterologous protection observed here thus likely reflects not only the hypervirulence and rapid systemic dissemination of type I parasites, but also their substantial antigenic divergence from the type II vaccine strain, which may limit the recognition of critical type I-specific epitopes by vaccine-induced T cells. These findings have important implications for future vaccine development. While PruΔ*ap2X-1* shows promise as a vaccine candidate against homologous and closely related strains, achieving broader cross-protection against hypervirulent type I strains may require alternative strategies, such as the inclusion of conserved or cross-reactive antigens capable of eliciting immunity across genetically diverse lineages, or the use of multivalent vaccine formulations incorporating immunodominant epitopes from multiple strain types. Collectively, our results demonstrate that PruΔ*ap2X-1* immunization provides robust protection against low-virulence strains and partial protection against the highly virulent RH strain, underscoring the necessity of incorporating genotype-spanning antigen designs in future vaccine development efforts.

Beyond protection against acute infection, immunization with PruΔ*ap2X-1* also conferred robust protection against chronic infection. All immunized mice survived oral challenge with 10 or 40 Pru tissue cysts; and importantly, no parasites were detected in their brains, as determined by both microscopic examination and *B1* gene amplification. This finding is particularly significant because the ability of *T. gondii* to convert from rapidly replicating tachyzoites to quiescent bradyzoites within tissue cysts is a key factor in its pathogenesis and transmission [37]. This developmental transition is tightly regulated by AP2 transcription factors, and its disruption represents a promising strategy to prevent the establishment of latent infection [22,25,31]. Although AP2X-1 has been reported to regulate stage conversion and sexual development [27], the present study did not directly investigate the biological consequences of its deletion on these processes. Based on our data, we can only conclude that immunization with PruΔ*ap2X-1* effectively prevents the establishment of chronic infection, as evidenced by the absence of detectable brain cysts and parasite DNA in vaccinated mice under the tested conditions. We speculate that the protection against chronic infection may involve the intrinsic cyst-forming defect of the vaccine strain and/or the vaccine-induced immune responses that suppress cyst development upon challenge. The relative contribution of each mechanism requires further study.

Characterization of humoral and cellular cytokine response revealed that PruΔ*ap2X-1* immunization elicited significantly elevated levels of *Toxoplasma*-specific total IgG, IgG1 and IgG2a, with IgG2a levels exceeding IgG1, indicating a predominantly Th1-biased humoral response [38]. The Th1-skewed response typically elicited by *T. gondii* is essential for host resistance to infection [38]. Notably, while IL-12 was not significantly elevated at 45 dpv in the present study, the PruΔ*gra72* vaccine—which also has a type II background—was previously reported to induce significant IL-12 upregulation at the same time point in the same mouse model [18]. This discrepancy may reflect differential effects of gene deletion on the activation of the IL-12/IFN-γ axis, or potential regulatory roles of AP2X-1 deletion on IL-12 secretion by dendritic cells or macrophages. Nevertheless, the marked upregulation of IFN-γ in PruΔ*ap2X-1*-immunized mice suggests that the peak of IL-12 secretion may have occurred at an earlier time point, while IFN-γ, as a downstream effector cytokine, remained elevated [39]; alternatively, immunomodulatory factors encoded by *T. gondii* may have partially suppressed sustained IL-12 production [40].

While Th1-type immunity is essential for controlling *T. gondii* infection, excessive Th1 inflammation can cause immunopathological damage [41,42]. The relatively moderate upregulation of Th2-associated cytokine IL-4 and the regulatory cytokine IL-10 observed in this study may represent a regulatory mechanism to limit Th1-driven inflammation [43], thereby balancing effective anti-parasitic immunity with host protection against tissue damage. Thus, the balanced Th1/Th2 response elicited by PruΔ*ap2X-1* immunization likely contributes to both effective protection and the favorable safety profile observed in this study [44]. It should be noted that, as a proof-of-concept study, this immune evaluation was designed primarily to assess the Th1/Th2 polarization trend induced by the vaccine candidate; therefore, the analysis was focused on serum antibodies and a selected panel of cytokines. More comprehensive cellular immune parameters—such as T cell subset differentiation, memory T cell markers, and T cell polyfunctionality—were not examined, representing a limitation of the present study. Systematic evaluation of these parameters will be performed in follow-up investigations to fully elucidate the mechanisms underlying PruΔ*ap2X-1*-induced protective immunity.

Several limitations of the present study should be acknowledged. The use of outbred Kunming mice introduces greater inter-individual variation, limiting comparability with inbred-strain data; moreover, the single time point (45 dpv) and absence of cellular immune profiling together restrict our assessment to short-term protection without mechanistic insight. Safety and virulence assessment of PruΔ*ap2X-1* relied primarily on survival curves and brain cyst counts, lacking quantitative organ parasite burden, histopathological evaluation, or behavioral observation. The minimum protective dose was not determined, and the detection limit of the semi-nested *B1* PCR assay was not established; therefore, caution is warranted when interpreting the negative PCR results. Protection against congenital and oocyst-mediated infection also remains unevaluated. More critically, several safety data—including evaluations in other host species (e.g., food-producing animals and cats), in immunocompromised hosts and pregnant animals, biodistribution and tissue persistence of the vaccine strain, as well as genetic stability and risk of reversion to virulence—remain entirely unavailable, representing major gaps in the current safety profile that must be systematically addressed before any clinical or field application. Accordingly, future studies should prioritize efficacy validation in food-producing animals, long-term immune memory assessment, comprehensive safety profiling, protection evaluation via natural infection routes, and practical translational aspects such as scale-up production and stability.

## 5. Conclusions

The present study demonstrates that the PruΔ*ap2X-1* knockout strain is markedly attenuated in mice, exhibiting 100% survival at the highest tested dose with no detectable brain cysts. Immunization with PruΔ*ap2X-1* confers robust protection against low-virulence *T. gondii* strains (type II Pru and ToxoDB#9 PYS), partial protection against the hypervirulent type I RH strain, and completely prevents chronic infection following tissue cyst challenge, with no detectable parasites in the brain under the experimental conditions. Furthermore, PruΔ*ap2X-1* immunization elicits elevated IgG2a levels and robust IFN-γ production, indicative of a Th1-biased immune response. Collectively, these findings support PruΔ*ap2X-1* as a promising candidate for a live-attenuated vaccine against toxoplasmosis. While further evaluation of PruΔ*ap2X-1* in other economically important species and congenital models is essential to assess its translational potential, the current findings remain provisional due to the lack of peripheral organ parasite burden data and the absence of comprehensive safety and cellular immune evaluations.

## Data Availability

The datasets supporting the findings of this article are included within the paper and its Supplementary Materials.

## Supplementary Materials

The following supporting information can be downloaded at: https://www.mdpi.com/article/10.3390/ani16162453/s1. Figure S1: IFN-γ levels significantly exceeded those of other cytokines (IL-2, IL-4, IL-10, and IL-12) at 45 dpv. Data are shown as individual values with median ± 95% CI. Statistical comparisons were performed using the Mann–Whitney U test: IFN-γ versus IL-2, *p* = 0.0079; IFN-γ versus IL-4, *p* = 0.0022; IFN-γ versus IL-10, *p* = 0.0022; IFN-γ versus IL-12, *p* = 0.0079; Figure S2: PCR detection of the *B1* gene in brain tissues of immunized mice following cyst challenge. Lanes A1–A10: samples from mice challenged with 10 cysts; lanes B1–B10: samples from mice challenged with 40 cysts. P: positive control; N: negative control; Table S1: Brain cyst burden and *B1* gene detection results of the surviving mice challenged with Pru cysts.
