# Supplementary material for: A Live Attenuated AP2X-1-Deficient Toxoplasma Strain Confers Protective Immunity Against Acute and Chronic Toxoplasmosis in a Murine Model
Source: Animals (Basel). 2026 Aug 7;16(16):2453. doi: 10.3390/ani16162453 (PMC13508968; doi:10.3390/ani16162453)
Supplement: Supplementary file 1 [file animals-16-02453-s001.zip › animals-4408508.R1-Table S1.pdf]

Table S1. Brain cyst burden and *B1* gene detection results of the surviving mice challenged with Pru cysts.

Group	Average brain cyst number	Positive rate of *B1* gene	Brain cyst number (*B1* gene detection result)									
			1	2	3	4	5	6	7	8	9	10
Naive +10 cysts	516.67	-	450	350	750	-	-	-	-	-	-	-
Immunized +10 cysts	0	0%	0 (-)	0 (-)	0 (-)	0 (-)	0 (-)	0 (-)	0 (-)	0 (-)	0 (-)	0 (-)
Immunized + 40 cysts	0	0%	0 (-)	0 (-)	0 (-)	0 (-)	0 (-)	0 (-)	0 (-)	0 (-)	0 (-)	0 (-)
